# Delayed union of a surgically treated fragility fracture of the pelvis: A case report

**DOI:** 10.1016/j.ijscr.2020.04.093

**Published:** 2020-05-12

**Authors:** Yohei Yanagisawa, Yukei Matsumoto, Tetsuya Hoshino, Yoshiaki Inoue, Masashi Yamazaki

**Affiliations:** a Department of Emergency and Critical Care Medicine, University of Tsukuba Hospital, 2-1-1 Amakubo, Tsukuba, Ibaraki, 305-8576, Japan; b Department of Orthopaedic Surgery, University of Tsukuba, 1-1-1 Tennodai, Tsukuba, Ibaraki, 305-8575, Japan; c Department of Emergency and Critical Care Medicine, University of Tsukuba, 1-1-1 Tennodai, Tsukuba, Ibaraki, 305-8575, Japan

**Keywords:** Delayed union, Non-union, Fragility fractures of the pelvic ring, Osteosynthesis, Case report

## Abstract

**INTRODUCTION:**

There are no universally accepted treatment strategies for fragility fractures of the pelvis (FFPs). The incidence of delayed union or non-union of Type IIIa FFP is still unknown.

**PRESENTATION OF CASE:**

We describe a case of delayed union of a Type IIIa FFP. A 96-year-old female patient who lives independently accidentally fell when exiting a car. The diagnosis of Type IIIa FFP with displaced left ilium and left pubic rami fracture. Surgical repair was performed using an anterior intrapelvic approach with constructs made using two reconstruction contoured plates to bridge the medial edge and middle part of the fracture. This case was revealed delayed union. The periodic CT examinations were performed to determine the progress of bone union. The patient returned to most social activities including living independently and the Modified Majeed score was 94 at 12 months post-operation.

**DISCUSSION:**

For the case of TypeⅢa FFP, soft tissue is spread over a wide area. When the fracture site of ilium was exposed, the cortical bone was found to be thin with poor blood flow. There is a possibility that the blood flow was hindered by subperiosteal elevation of the iliacus muscle from the internal iliac fossa in this case.

**CONCLUSION:**

Non-union of the iliac wings is relatively rare following high-energy pelvic ring fractures. The incidence of delayed union or non-union of Type IIIa FFP remains unknown; therefore, careful follow-up of patients who undergo treatment is necessary to reduce the risk of delayed union.

## Highlights

• A case of delayed union of type IIIa fragility fracture of the pelvis.
• Evaluating the progress of bone fusion may be difficult using front and side X-rays.
• This may be because of the implant position and pelvis shape.
• CT enables diagnosis of delayed union and non-union in type IIIa pelvis fracture.

## 1 Introduction

Fragility fractures are caused by low-energy accidents such as falls from either a standing position or a low height. Approximately 90 % of fragility fractures occur in female individuals, primarily those aged 58–75 years, such fractures are not uncommon among older individuals [1]. The most common sites of fragility fractures are the vertebra, proximal femur, distal radius and proximal humerus [1]. With increasing life expectancy, fragility fractures of the pelvic ring (FFPs) are becoming increasingly frequent.

In 2013, Rommens suggested a classification system for FFP [2]. The system is based on injury localization and the presence of fracture displacement in order to categorize FFPs into four major types (Type I to IV) and several subtypes. Clinical and radiological criteria are routinely used to characterize FFP and to evaluate the proper treatment; typically, for Type I FFPs, no surgical therapy is needed, while percutaneous screw fixation is recommended for Type II FFPs [2]. While Type III and IV FFPs typically required open reduction and internal fixation is required, the decision to undergo operative treatment is still somewhat subjective [2]. Rommens reported Type IIIa FFPs to account for 8.2 % of the total population (20 out of 245 cases of FFP) [2]. In such fractures, the posterior fragment of the ilium is translocated, and if conservative treatment is carried out, the revalidation time will be longer and more problematic than for Type I or II FFPs. Dislocations of iliac fractures are large, therefor often require anterior intra pelvic approach for open reduction and osteosynthesis was needed. The incidence of delayed union or non-union of Type IIIa FFP is still unclear, because the clinical features of such cases have not been sufficiently reported to date. Rommens suggested that further clinical and biomechanical investigations are needed to determine optimal treatment strategies for these pelvic lesions [3].

We present a case of delayed union of Type IIIa FFP. The fracture healing process was followed up in detail through computed tomography (CT) examination as fracture healing is difficult to evaluate using standard X-rays. As there are no widely accepted fixation methods for FFPs, we aim to present a case report that may inform the decision to undertake operative treatment for Type IIIa FFPs. The following case report is compliant with SCARE guidelines [4].

## 2 PRESENTATION OF CASE

**Fig. 1:**
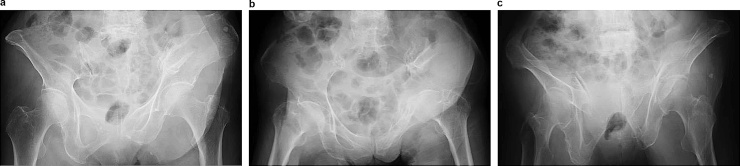
Representative pre-operative X-ray images taken during admission for fragility fracture of the pelvic ring. (a) Pre-operative pelvic anteroposterior view. (b) Pre-operative pelvic inlet view. (c) Pre-operative pelvic outlet view.

A 96-year-old woman, 140 cm in height and 30 kg in weight, presented with a pelvic fracture due after falling from a standing height when getting out of the car. She was unable to walk and was transported to an adjacent hospital by ambulance, after which she was transferred to our hospital for surgical treatment of FFP. Upon admission to our hospital, the patient was hemodynamically stable and moderately healthy with minor comorbidities (chronic gastritis, insomnia and osteoporosis). The patient was not taking any medication for osteoporosis at the time of injury as she did not want treatment. The diagnosis of Type IIIa FFPs, displaced left ilium and left pubic rami fracture was made by X-ray and CT (Fig. 1).

**Fig. 2:**
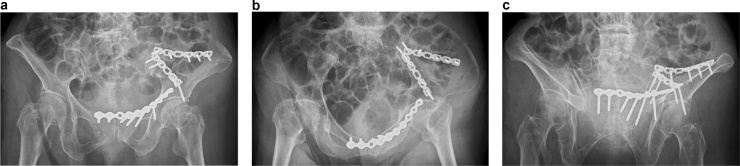
Representative post-operative X-ray images taken following surgical repair of fragility fracture of the pelvic ring. (a) Post-operative pelvic anteroposterior view. (b) Post-operative pelvic inlet view. (c) Post-operative pelvic outlet view.

The fracture was classified as American Society of Anesthesiologists physical status class 1, and the Charlson Comorbidity Index was 0. Pre-operative blood tests revealed anemia (hemoglobin level, 8.2 g/dL). Bone mineral density of the femoral neck was 0.316 g/cm^2^ and the young adult mean score was 40 %. Open reduction and internal fixation was performed 4 days after the injury under general anesthesia with the patient in the supine position. An anterior intrapelvic approach (modified Stoppa with a lateral window of the ilioinguinal approach) was used and reduction of the fracture gap was assessed under direct vision intraoperatively. The third bone fragment of iliac was not able to be fixed with the implant due to dislocation to the dorsal side from fracture site. Later, this site exhibited delayed union. Constructs were made using two reconstruction contoured plates (Locking Compression Plate [LCP®] 3.5 mm, Synthes) bridging the medial edge and middle part of the fracture, with bi-cortical screws using the locking holes where possible (Fig. 2). The surgery time was 3 h and 10 min, and intraoperative blood loss was 200 mL. In the post-operative period, 560 mL of red cell concentrate was transfused due to anemia. For safety reasons, the patient was transferred to the high-care unit for monitoring; intravenous hydration was withdrawn and oral food intake was allowed 6 h post-operatively. Weight bearing was not allowed for 6 weeks post-operatively, after which the patient was allowed to progress to full weight bearing.

**Fig. 3:**
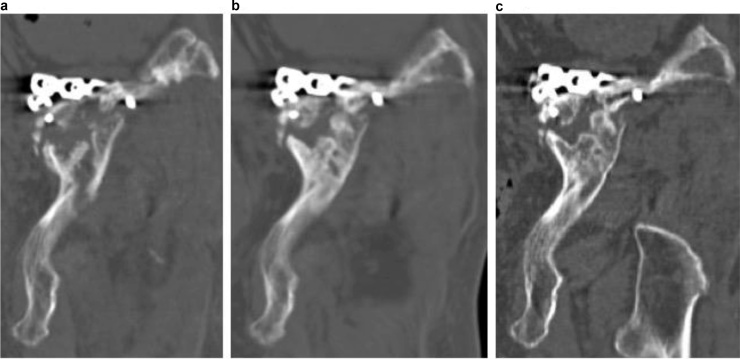
Post-operative computed tomography coronal views. Computed tomography images showing the process of union of the fracture site at (a) 5, (b) 12 and (c) 19 months after surgery reveal that union occurred between 12 and 19 months post-operatively.

**Fig. 4:**
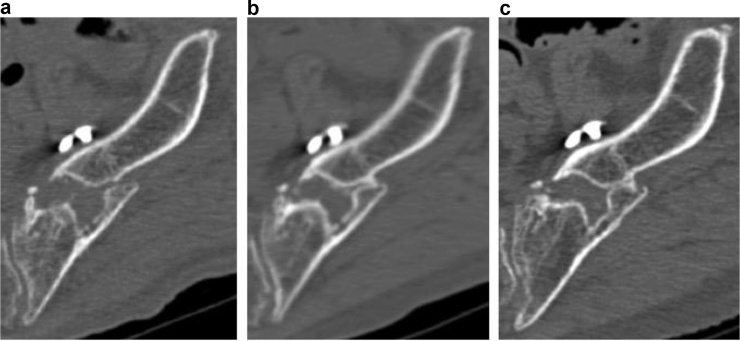
Post-operative computed tomography axial views. Computed tomography images showing the process of union of the fracture site at (a) 5, (b) 12 and (c) 19 months after surgery reveal that union occurred between 12 and 19 months post-operatively.

**Fig. 5:**
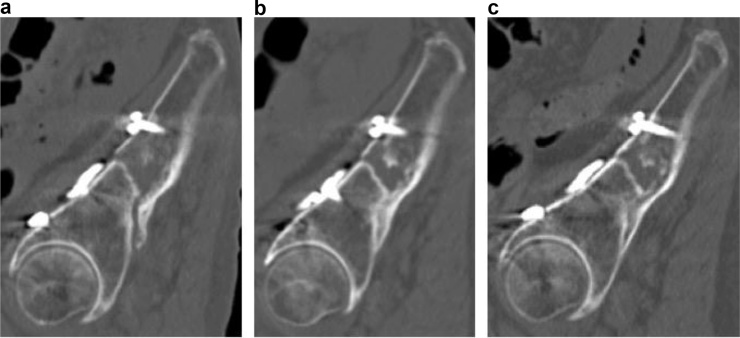
Post-operative computed tomography sagittal views. Computed tomography images showing the process of union of the fracture site at (a) 5, (b) 12 and (c) 19 months after surgery reveal that union occurred between 12 and 19 months post-operatively.

Evaluation of bone union by pelvic X-ray (anteroposterior, inlet and outlet view) was difficult because the plates, screws and fracture overlapped. Therefore, periodic CT examinations (post-operative 3, 5, 12 and 19 months) were performed to determine the progress of bone union. This revealed delayed union of the fracture (Fig. 3, Fig. 4, Fig. 5).

The patient experienced no pain waking with a walking stick and returned to most social activities including living independently within 6 months of the operation. The Modified Majeed score was 94 (except sexual intercourse, which was 4 points out of a possible 96) at 12 months post-operation.

## 3 Discussion

We present a case of delayed union of a Type IIIa FFP. Without the use of CT, the progress of bone union and identification of delayed union would be difficult. In the present case, we were able to observe the progress of union of the fractured part using regular CT, and thus present—to the best of our knowledge—the first detailed account of delayed union monitored through CT. Previous studies have reported the delayed union or non-union of iliac FFPs [[5], [6], [7]]. Non-union of the iliac wings is relatively rare following high-energy pelvic ring fractures [[5], [6], [7]] owing to the large metaphyseal bony contact available for healing [6]. It may be necessary to carry out careful examination of the post-operative bone union process in fragility iliac wing fractures in cases similar to the present case. It is difficult to evaluate only with X-ray, and the fact that this is the first report of evaluation by CT may explain why only there have only been a few reports of the process of bone union in the case of Type IIIa FFPs. In these fractures, it may be difficult to assess the progress of bone fusion from front and side X-rays due to the position of the implant and the shape of the pelvis. Using CT, especially multiplanar reconstruction (MPR), enables easy diagnosis of delayed union and non-union and evaluation of fracture healing even with the remaining metal hardware [8,9]. Examination of the fracture site with MPR in the present case allowed close examination of the status of the bridge callus and bone union.

During follow-up, we observed delayed union of the iliac wing in the context of FFP. Fracture union of iliac wing fractures in FFPs is generally expected to be good, but careful follow-up is necessary. Careful follow-up may be necessary for patients with findings such as prolonged pain around fracture site. Such cases may include delayed union and non-union. Late recognition of these may lead to implant breakage. As was used in the present case, CT imaging can be used to assess fracture status and may be useful during follow-up after surgical treatment of Rommens Type IIIa fractures.

We used a modified Stoppa with a lateral window of the ilioinguinal approach in the present case. The lateral window exposed by subperiosteal elevation of the iliacus muscle from the internal iliac fossa allows exposure of the iliac crest and the internal iliac fossa medially to the sacroiliac joint and distally to the pelvic brim. This reduces intraoperative bleeding from the lateral window [10,11]. When the fracture site of the present case was exposed, the cortical bone was found to be thin with poor blood flow. Considering the diamond concept [12], there is a possibility that the blood flow was hindered by subperiosteal elevation of the iliacus muscle from the internal iliac fossa in this case. Bone union is more prolonged in the elderly than in the young [12]. For this reason, in the case of FFP type IIIa, soft tissue is spread over a wide area, and the periosteum is separated from the iliac bone and fixed with a plate, as in the case of iliac fractures due to high-energy trauma. Percutaneous fixation may need to be considered in the future rather than the surgical method used for plate fixation, like in the present case.

## 4 Conclusion

Non-union of the iliac wings is relatively rare following high-energy pelvic ring fractures. The incidence of delayed union or non-union of Type IIIa FFP remains unknown; therefore, careful follow-up of patients who undergo treatment for Type IIIa FFPs is necessary to reduce the risk of delayed union. The use of CT, particularly multiplanar reconstruction, enables easy diagnosis of delayed union and non-union of Type IIIa FFPs.

## Declaration of Competing Interest

None.

## Funding

None.

## Ethical approval

Due to the retrospective nature of this study, ethical approval was not required.

## Consent

Written informed consent was obtained from the patient for publication of this case report and accompanying images. A copy of the written consent is available for review by the Editor-in-Chief of this journal on request.

## Author contribution

YY performed the operation and obtained informed consent for this report.

YY, YM, and TH took care of the patient during the hospitalization.

YI and MY critically revised the manuscript.

All authors have read and approved the final version of this manuscript.

## Registration of research studies

NA.

## Guarantor

Yohei Yanagisawa.

## Provenance and peer review

Not commissioned, externally peer-reviewed.
